# Participation Beyond Compliance: Who Tried to Influence Other People's Vaccination Behaviour During the COVID‐19 Crisis?

**DOI:** 10.1111/1467-9566.70170

**Published:** 2026-03-08

**Authors:** Hugo Touzet, Benoît Giry, Jeremy K. Ward

**Affiliations:** ^1^ Université Paris Cité CNRS Inserm Villejuif France; ^2^ Sciences Po Rennes ‐ Arènes (UMR CNRS 6051) Rennes France

**Keywords:** conflict avoidance, COVID‐19, participation, politicisation, vaccination

## Abstract

In this paper, we call for more attention to be paid to what we call ordinary contributions to public health policies: the propensity of ordinary citizens to actively influence others to follow or reject a health policy. Shifting the focus from personal compliance to active participation (i.e., ordinary contribution) raises distinct questions pertaining to self‐empowerment, personal network composition and the public denunciation of antivaccinationism. We draw on a survey conducted during the summer of 2022 among a representative sample of the French public (*n* = 4004) to understand what made some people try to bear on other people's behaviours regarding COVID‐19 vaccination. We asked respondents whether they tried to convince people in their various social circles to get vaccinated or to not get vaccinated. We found that a significant share of French people—especially the most vaccine‐hesitant—kept their opinions to themselves. Controlling for vaccine hesitancy and concern regarding COVID‐19, the propensity to engage in ordinary contributions was heavily influenced by relationship to politics. We discuss the overlapping between political competence and health literacy and the tensions that can arise in everyday discussions of issues at the interface of health and politics.

## 1 Introduction

In this paper, we will use the case of COVID‐19 vaccination in France to analyse public dispositions to engage in what we will call *ordinary contributions to public health policies*: the propensity to go beyond personal compliance (or noncompliance) and try to influence other people's health behaviours.

We define *ordinary contributions to public policies* as the effort to influence other people's behaviours undertaken by actors outside an institutional setting (profession, mandate, position in an organisation, social movement collective…) and performed via interactions taking place in everyday social exchanges (Giry 2023). *Ordinary contributions to public policies* are a specific facet of what James Coleman defined as « intervening activities »: the occurrences when people intervene in public settings to enforce a norm and contribute to a public good (Coleman 1990). *Ordinary contributions to public policies* are, therefore, a form of political engagement distinct from the various actions centred on (a) partisan competition and elections, (b) public participation initiatives set up by the State and other institutions and (c) social movement mobilisations. *Ordinary contributions to public policies* are a specific type of intervening activity which engages with a very particular type of norm: one that is promoted by the State through a public policy, such as vaccination against COVID‐19.

As for most public health policies, the success of a vaccination campaign rests on the ability of policymakers to enrol the public and elicit certain behaviours. A tremendous number of studies have been devoted to understanding how people form their personal opinions and the determinants of their behaviours on issues such as vaccination (Brewer et al. 2007; Dubé et al. 2021).

However, one limit of the existing literature on the enrolment (or lack thereof) of citizens in public policies is that most studies, including our own, focus on attitudes and behaviours taken in isolation (for exceptions, see Are et al. 2024; Brunson 2013; Facciani et al. 2023). They rarely consider people as potentially active in trying to influence people around them, even though they often show that people can be heavily influenced by interactions with their close ones. Indeed, during the pandemic, anyone could see in their daily life that personal beliefs were regularly transformed into attempts to convince others. Although some did so in a professional context (such as health professionals) or as part of social movements (patient associations, antivaccine collectives etc.) or other forms of policy entrepreneurship, we would like to focus on the more ordinary and less institutionalised forms of participation in public health policies that take place in the fabric of everyday life.

Shifting the focus from personal compliance to active participation (i.e., *ordinary contributions*) raises new and distinct questions. The COVID‐19 pandemic has put vaccination at the centre of people’s lives as well as ushered in increased politicisation of discussions around vaccination in many countries. Political identities (ideology, values, partisanship), therefore, bear heavily on the way people form their beliefs regarding vaccination, as was well documented in recent years (for reviews of the literature, see Motta 2021; Wagner and Eberl 2024). Another implication is that, to go beyond belief formation and compliance, it is necessary to engage with other strands of political sociology and especially work on the manifold reasons why so many people keep their political opinions to themselves. Consequently, turning attention towards what we call *ordinary contributions to public health policies* contributes to understanding health behaviours both by setting people within their social environment and interactions as well as by broadening the scope of political dispositions taken into account.

To advance the understanding of what drives some people to engage in such ordinary contributions to public policies, we focus on vaccination against COVID‐19. We draw on a survey conducted during the summer of 2022 among a large sample of the French public (*n* = 4004). We asked respondents whether they tried to convince people in their various social circles to get vaccinated or to not get vaccinated. In the next section, we formulate two sets of hypotheses explaining differences in propensity to try to influence other people’s vaccination behaviours: one anchored in people’s relationship to health, the other in their relationship to politics.

## 2 Determinants of Ordinary Contributions to COVID‐19 Vaccination: Guiding Hypotheses

### 2.1 Health, Vulnerability to Diseases and Perceptions of Vaccines

When governments try to convince their constituents to get vaccinated, they usually focus on two types of arguments: the capacity of vaccines to protect the person’s health and their capacity to prevent transmission to others. Much of the communication around vaccines emphasises the latter and bolsters values of solidarity and participation in the collective good. The implications of this for individual motivations to vaccinate have been well theorised using a broad understanding of self‐interest by economists working on private contributions to public goods (Andreoni 1990).

It results from this that the propensity to engage in ordinary contributions to vaccination must depend on one’s belief that the vaccine will produce this private and collective good. We can, therefore, expect that this propensity depends on the same determinants as attitudes to vaccines, the first of which are the perceptions of epidemic danger and the vaccine’s efficacy and safety (Brewer et al. 2007; Dubé et al. 2021; Larson et al. 2022). In the case of COVID‐19, we should, therefore, see positive contributions from those who feel more at risk of the disease, such as those most worried about the pandemic generally (Larson et al. 2022). Opposite them, those who believe the vaccines to be unsafe would be more likely to try to convince their loved ones to refuse the vaccine because they want to protect them from its purported risks.

This dimension leads to a first set of two hypotheses:

H 1.1 Positive ordinary contributions to COVID‐19 vaccination will be more prevalent among those who are (or feel) more vulnerable to the disease.

H 1.2 Negative ordinary contributions to COVID‐19 vaccination will be more prevalent among those who are more hesitant towards these vaccines.

Of course, the clear separation between the two is somewhat artificial and simplistic because perceived vulnerability to the disease is usually associated with less reticence. It is also important to note that studies have consistently shown that many people are both vaccine‐hesitant and worried about vaccine‐preventable diseases (Enkel et al. 2018) (Ward, Privault, et al. 2024). These two hypotheses, and the ones that will come after, are, therefore, meant to help guide the analysis and to facilitate the understanding of how these different factors can intersect.

However, there is a crucial difference between holding beliefs and acting upon these beliefs, as the literature on vaccine hesitancy has consistently shown (Dubé et al. 2021; Larson et al. 2022).

In the present case, there is good reason to think that not everyone will tell the people around them what they think they should do. To engage in any form of ordinary contribution to COVID‐19 vaccination requires a sense of competence and self‐empowerment on health issues but also a degree of interest in such issues. In France, as in most countries, gendered socialisation entails that women take on disproportionately the tasks pertaining to healthcare and care more generally. Women are more likely to be the main decision‐makers regarding the vaccination of their children and to discuss this issue among their peers (Berezin and Eads 2016; Reich 2016). A third hypothesis is, therefore, that…

H 1.3 Women will be more likely to engage in all types of ordinary contributions to COVID‐19 vaccination.

### 2.2 Political Attitudes and Ordinary Contributions to Vaccination

Studies have also shown that a wide variety of aspects of relationships to politics can influence people’s attitudes to vaccines. For instance, vaccine hesitancy has been found to be more prevalent among people who feel close to parties at the far ends of the political spectrum and to populist parties (Kennedy 2019; Merkley 2020) (Ward, Cortaredona, et al. 2024), who identify as conservatives (Motta 2021), who distrust the government and other political actors (Denemark et al. 2022; Spälti et al. 2023) (Ward, Cortaredona, et al. 2024) and who have a low level of political sophistication (Blank and Shaw 2015; Enders and Uscinski 2021) (Ward, Privault, et al. 2024). Much research has also investigated how these different dimensions can combine to produce vaccine hesitancy (Blank and Shaw 2015; Choi and Fox 2022; Enders and Uscinski 2021; Merkley 2020; Motta 2021) (Ward, Cortaredona, et al. 2024) as well as the issues pertaining to the depoliticisation of vaccination (Numerato et al. 2021). Interestingly, this literature at the interface between sociology, political science and cognitive sciences has mapped out a significant number of sociocognitive mechanisms via which dispositions towards health and politics affect the formation of beliefs on vaccines. For instance, a major current debate regarding partisan differences in attitudes to vaccines opposes explanations based on (a) motivated reasoning—the idea that through a variety of cognitive processes and biases, people select and interpret information differently depending on their prior political worldviews—to explanations based on (b) Bayesian inference—the idea that people come to different conclusions not because they treat information differently but because they have different information regimes that lead them rationally to different conclusions (Joslyn and Sylvester 2019; Motta 2021).

However, the fact that vaccines become part of public debates and political news does not just affect belief formation. It also has consequences regarding how vaccines are discussed in everyday life. It entails that they can be discussed as a political issue and, therefore, that several aspects of relationships to politics can bear on people’s disposition to engage in *ordinary contributions to vaccination*.

Firstly, the framing of vaccination as a government policy put forward by representatives of a given political party entails that the disposition towards publicly supporting it is likely to be influenced by the proximity towards said political party. Rejection of the governing party can affect the judgement on the vaccines themselves. More importantly for the question at the core of this paper, such rejection can dissuade people from publicly supporting this policy because it might provide the opposing group with symbolic and material resources or because of fear of reputational costs. Symmetrically, rejection of the governing party can constitute a further incentive to convince people not to be vaccinated: Vaccination can become one of the issues via which people try to convince others to become more politically aligned with them. The same reasoning applies to a second important and multifaceted aspect of relationships to politics: trust in political actors and institutions (Norris 1999). People who have a high degree of political distrust can be more reticent to express support for a public policy, but they can also be less likely to take an interest in such a policy because of its origin in « mainstream politics ».

H 2.1 People who are closer to the governing party will be more likely to contribute positively to COVID‐19 vaccination, whereas those who are farther are more likely to contribute negatively.

H 2.2 People with a high degree of political distrust will be more likely to contribute negatively to COVID‐19 vaccination, whereas people with a high degree of political trust will be more likely to contribute positively.

The third and most important dimension is political sophistication, which encompasses political engagement and competence (Gamson 1992; Zaller 1992). Classical work in political science has shown that many people are disinterested in politics and feel incompetent or illegitimate to have an opinion on many political issues (Bourdieu 1979; Converse 2006). The fact that vaccination made its way into the news and into political debates means that interest in this issue can develop via interest in politics and the social practices that support it. Studies have already shown that political sophistication can be a determinant of attitudes to vaccines (Blank and Shaw 2015; Enders and Uscinski 2021; Joslyn and Sylvester 2019) (Ward, Cortaredona, et al. 2024). Political sophistication can also facilitate the public expression of attitudes to vaccines by favouring interest in vaccination and the feeling that one is competent on the subject. Also, the fact that vaccination becomes a political topic expands the types of social situations where it is likely to be discussed, creating more opportunities to engage in *ordinary contributions to public health policies*.

H 2.3 The more politically sophisticated will be more likely to engage in ordinary contributions to COVID‐19 vaccination.

## 3 Context of the Study and Methods

### 3.1 Vaccination Against COVID‐19 in France

COVID‐19 emerged at a difficult time for vaccination in France. During the 2000s, vaccine controversies multiplied, and France was identified as one of the countries where negative attitudes towards vaccines were among the most widespread (Larson et al. 2016). The epidemic also emerged at a time of transformation in the political arena. Since 2017, there has emerged an opposition between, on the one hand, a new party presenting itself as centrist (La République En Marche/Renaissance, founded by Emmanuel Macron, in power since 2017) and, on the other hand, a far‐right party (the Rassemblement National, led by Marine Le Pen) and a party positioned more towards the radical left (France Insoumise, founded by Jean‐Luc Mélenchon). La République En Marche made the extension of compulsory vaccination one of its flagship policies in 2017, and several leading representatives of the Rassemblement National and France Insoumise expressed hesitation about vaccines and vaccination requirements during that period and the pandemic (Ward, Cortaredona, et al. 2024).

The COVID‐19 vaccination campaign began in France at the end of December 2020 and gradually expanded until it covered all adults in May 2021, followed by children. Surveys carried out just before the start of the campaign showed that reluctance towards this future vaccination campaign was very prevalent. However, the share of French adults who intended to get a vaccine or were already vaccinated improved significantly up to the summer of 2021 (Ward, Privault, et al. 2024). Despite this improvement in attitudes, the government decided, in early July 2021, to introduce a Health Pass, making it very costly not to vaccinate. This decision sped up the increase in two‐dose vaccination coverage. In February 2022, the Health Pass, which became the « vaccination pass », was repealed. Our survey, conducted almost six months later, came at a time when lockdowns were in the distant past and when most political and media actors had moved their attention away from COVID‐19 and health more generally.

### 3.2 Data

We draw on a survey conducted online between July 12th and August 9th, 2022, among a sample designed to match the composition of the French adult mainland population (*n* = 4004), randomly selected from the main research panel used by researchers in France (coordinated by the company Bilendi SA). We aimed to approach representativeness via quota sampling (age, gender, occupation, population size of the area of residence and region; see Table 1 for a description of the sample and Supporting Information Table S1 for a comparison with official census data). An email was sent to 80,076 participants on the panel, and data collection was stopped when quotas were reached, resulting in a very good fit with official census data on these quotas (± 2 percentage points maximum). The survey contained a number of questions pertaining to the COVID‐19 epidemic, vaccination and relationships to politics. Respondents could not skip questions but were allowed to select “don’t know” or “prefer not to say” for most questions. The methodology of the study was reviewed and approved by the Institutional Review Board of the INSERM (decision n°21–770).

**TABLE 1:** Factors associated with contributing to COVID‐19 vaccination, bivariate analysis, row %.

	*N*	Noncontributors (27.6%)	Versatiles (13.9%)	Allies (49.5%)	Opponents (9.0%)	Statistical test
Age (*p* < 0.001)						Pearson’s *X*²
18–24	401	26.4%	22.2%	40.1%	11.2%	
25–34	543	29.3%	23.0%	32.2%	15.5%	
35–49	894	33.2%	17.1%	39.4%	10.3%	
50–64	1033	29.5%	10.6%	52.5%	7.4%	
65–74	620	21.0%	8.1%	64.4%	6.6%	
75 and over	513	20.9%	6.0%	68.4%	4.7%	
Gender (*p* = 0.004)						Pearson’s *X*²
Male	1868	27.2%	15.7%	49.3%	7.9%	
Nonmale	2136	27.9%	12.4%	49.6%	10.1%	
Monthly income (*p* < 0.001)						Pearson’s *X*²
1500–2000	633	32.4%	15.8%	40.8%	11.1%	
Less than 1000	304	34.9%	19.1%	36.8%	9.2%	
1000–1500	477	31.0%	17.0%	39.8%	12.2%	
2000–3000	893	26.0%	13.7%	50.3%	10.1%	
3000–4000	722	25.1%	11.9%	55.1%	7.9%	
4000 and more	521	14.8%	11.9%	68.5%	4.8%	
Prefer not to answer	454	34.1%	10.8%	47.6%	7.5%	
Occupation (*p* < 0.001)						Pearson’s *X*²
Manual workers	462	39.2%	13.9%	30.3%	16.7%	
Farmers	30	26.7%	16.7%	53.3%	3.3%	
Self‐employed and entrepreneurs	150	28.7%	25.3%	36.0%	10.0%	
Senior executive professionals	450	19.1%	17.1%	56.4%	7.3%	
Middle executive professionals	582	31.3%	14.3%	44.8%	9.6%	
Employees	602	31.2%	15.8%	39.7%	13.3%	
Retirees	1321	21.4%	8.9%	64.5%	5.2%	
Never worked and others	407	32.7%	19.4%	40.3%	7.6%	
Formal education (*p* < 0.001)						Pearson’s *X*²
High school	1441	29.5%	14.0%	46.0%	10.5%	
No diploma	240	31.7%	28.3%	30.8%	9.2%	
Vocational secondary	738	32.7%	15.7%	41.6%	10.0%	
High school+2	746	26.4%	11.9%	53.9%	7.8%	
High school+3	400	22.5%	8.2%	62.0%	7.2%	
High school+4 or more years	439	17.1%	11.4%	65.1%	6.4%	
Worry level (*p* < 0.001)						Pearson’s *X*²
0 to 3	694	41.5%	7.5%	29.3%	21.8%	
4–6	1002	33.0%	14.5%	42.2%	10.3%	
7–8	1543	20.5%	15.9%	58.6%	4.9%	
9–10	650	17.2%	14.6%	64.6%	3.5%	
Do not know	115	48.7%	17.4%	26.1%	7.8%	
COVID‐19 vaccine hesitancy (*p* < 0.001)						Pearson’s *X*²
Q1	941	10.8%	6.9%	81.8%	0.4%	
Q2	688	16.7%	12.5%	69.8%	1.0%	
Q3	786	25.3%	18.2%	55.0%	1.5%	
Q4	843	39.5%	24.0%	27.9%	8.7%	
Q5	746	47.6%	8.3%	8.4%	35.7%	
Party identification (*p* < 0.001)						Pearson’s *X*²
Far left	627	24.2%	28.4%	37.0%	10.4%	
Left	268	19.0%	11.6%	66.8%	2.6%	
Green party	230	24.8%	9.1%	60.9%	5.2%	
Centre	717	13.0%	9.9%	74.3%	2.8%	
Right	282	18.4%	9.6%	67.0%	5.0%	
Far right	936	33.7%	13.5%	36.0%	16.9%	
No partisan proximity	837	42.1%	9.3%	39.4%	9.2%	
Other	107	29.9%	24.3%	37.4%	8.4%	
Political trust (*p* < 0.001)						Pearson’s *X*²
Q1	826	39.3%	10.2%	30.8%	19.7%	
Q2	788	34.0%	11.8%	40.4%	13.8%	
Q3	871	26.5%	12.9%	54.5%	6.1%	
Q4	733	22.0%	13.2%	61.8%	3.0%	
Q5	786	15.1%	21.9%	61.1%	1.9%	
Political sophistication (*p* < 0.001)						Pearson’s *X*²
Q1	821	43.7%	13.3%	30.0%	13.0%	
Q2	803	32.8%	13.8%	42.6%	10.8%	
Q3	779	23.9%	19.4%	48.4%	8.3%	
Q4	847	17.7%	11.3%	64.8%	6.1%	
Q5	754	19.4%	12.1%	61.8%	6.8%	

### 3.3 Dependent Variables

We asked respondents the following question: « Since the start of the COVID‐19 vaccination campaign, have you tried to convince people of the importance of getting vaccinated? ». Respondents had to say whether they had done so with « People in your close family circle (spouse, child, parent) », « People in your extended family circle (not living with you) », « friends », « Colleagues at work » and « People on social media or the Internet ». Response options were « very often », « often », « sometimes », « never », « I am not concerned » and « I do not wish to answer ». Using the same item structure, we also asked respondents « and have you tried to convince people that it was NOT important to get vaccinated? ». Responses to each question were turned into three outcome categories: « yes » (« very often », « often » and « sometimes »), « no » (« never ») and « not concerned » (« I am not concerned » and « I do not wish to answer »).

We analyse the patterns of responses to all questions taken together, thus identifying those who engaged only in positive contributions (*allies*), only in negative contributions (*opponents*), those who were *versatile* (sometimes positive, sometimes negative) and those who did not engage in contributions (*noncontributors*). Our main outcome will be this typology of four dispositions towards ordinary contributions to COVID‐19 vaccination policies.

### 3.4 Independent Variables

COVID‐19 worry (H1.1): We use the answers to the following question as our main proxy for perceived vulnerability to COVID‐19: “On a scale of 0–10, how worried were you about the impact of the COVID‐19 epidemic on your health or that of your loved ones?”. Following Peretti‐Watel’s fine‐grained analyses of the relationship between COVID‐19 worry and a number of outcomes during the pandemic, which showed significant differences among the most worried (Peretti‐Watel 2022), we divided the sample into the following five groups: 17.3% of respondents gave 0, 1, 2 or 3 as an answer; 25% gave 4, 5 or 6; 38.5% gave 7 or 8; 16.2% gave 9 or 10; and 2.9% responded “I don’t know”.

COVID‐19 vaccine hesitancy score (H1.2): The measures we used to assess attitudes to COVID‐19 vaccination reflect the fact that the survey was conducted well after the French government implemented a Health Pass making this vaccination almost mandatory. This policy enabled the attainment of a very large vaccine coverage (> 93% of adults had had at least one dose at the time of the survey) but also meant that many were vaccinated even though they were still reticent (Ward, Privault, et al. 2024). We measured attitudes to COVID‐19 vaccines using three questions targeting different aspects of COVID‐19 vaccination: « Do you still have doubts or reticence towards the Covid‐19 vaccine that you received ? A lot, a little, not really, not at all, don’t know, do not wish to respond »; « In the future, if it were recommended to vaccinate against Covid‐19 again to reinforce or prolong the protection against this disease, would you get this «booster» ? Yes, certainly; Yes, Probably; No, probably not; No, certainly not »; and « Do you agree with the following statements … It was useless to vaccinate healthy adults against Covid‐19: Totally agree, Somewhat agree, Somewhat disagree, Totally Disagree, Don’t know ». We performed a factor analysis on these 3 items (Cronbach’s alpha: 0.78) to calculate a COVID‐19 vaccine hesitancy score and divided respondents into five quintiles, with Q1 comprising the least vaccine‐hesitant and Q5 the most vaccine‐hesitant.

Party identification (H2.1): We asked respondents to which French political party they felt the closest (among a list of 17 parties) with the possibility to answer “another party” or “no party at all”. Responses were encoded into eight categories: far left, green party, left, centre, right, far right, no party and another party.

Political trust score (H2.2): Using the same method as for the COVID‐19 vaccine hesitancy score, we calculated a political trust score based on the level of trust (a lot, some, not much, not at all, do not know) attributed by respondents to four institutions central to political life in France: « the Government », « political parties », « the Parliament » and « the media » (Cronbach’s alpha: 0.77; Q1 = low trust, Q5 = high trust).

Political sophistication score (H2.3): We built a political sophistication score drawing on the three most frequently used items in French studies (Tiberj 2004): « would you say you are interested in politics? Yes, a lot; Yes, a litle, No, not really; No, not at all »; « how often do you follow political news? Everyday or almost; Several times a week; once or twice a week; Less often; Never »; and « how often do you partake in elections? You vote at every election; You vote at most elections; You vote at some elections; You rarely vote; You don’t vote ». Our score has a Cronbach’s alpha of 0.75 (Q1: low political sophistication, Q5 = high political sophistication).

Control variables: We asked respondents their age and divided the sample into 6 categories (18–24, 25–34, 35–49, 50–64, 65–74, 75+). Respondents were also asked whether they identified as male, female or other (H1.3). Only 0.2% responded “other”, so we created a dichotomous variable following common practice (male/nonmale). We also recorded respondents’ monthly income and their highest diploma and assessed respondents’ occupation following standard French nomenclatures (see Supporting Information Table S1).

We computed generalised variance inflation factors (GVIF) to assess potential multicollinearity. All variables presented GVIF^(1/(2 × Df)) values below 2.0, with most below 1.5, indicating no concerning multicollinearity (Supporting Information Table S6).

### 3.5 Data Analysis

To test our hypotheses, we study the correlations between belonging to each of these groups and a number of independent variables related to our hypotheses (see below). We use bivariate analyses (Pearson’s chi‐squared tests) and a multinomial regression model. We ran the same model four times, each time changing the reference group to facilitate interpretation (see Table 2 and Supporting Information Tables S2–S4). We apply Type II ANOVA tests to assess the contribution of each variable to the model. Type II ANOVA evaluates the unique effect of each predictor while controlling for the others (Fox and Weisberg 2019) (see Supporting Information Table S5). For the regression analyses in the Results section, effects are interpreted with the other variables in the model held constant.

**TABLE 2:** Factors associated with being a noncontributor, multinomial regression.

	Ref: Noncontributors					
	RRR	95% IC	RRR	95% IC	RRR	95% IC
	Versatiles		Allies		Opponents	
Age						
18–24	Ref	Ref	Ref	Ref	Ref	Ref
25–34	0.79	0.51, 1.23	0.75	0.50, 1.14	0.84	0.50, 1.40
35–49	0.53^**^	0.35, 0.81	0.72	0.49, 1.05	0.48^**^	0.29, 0.79
50–64	0.29^***^	0.19, 0.46	0.90	0.62, 1.32	0.41^***^	0.24, 0.69
65–74	0.18^***^	0.09, 0.36	0.84	0.47, 1.49	1.21	0.48, 3.04
75 and over	0.11^***^	0.05, 0.24	0.63	0.35, 1.14	1.10	0.40, 3.03
Gender						
Male	Ref	Ref	Ref	Ref	Ref	Ref
Nonmale	0.96	0.76, 1.22	1.36^**^	1.12, 1.65	1.21	0.91, 1.61
Monthly income						
1500–2000	Ref	Ref	Ref	Ref	Ref	Ref
Less than 1000	0.90	0.57, 1.45	1.09	0.73, 1.63	0.86	0.50, 1.49
1000–1500	0.88	0.59, 1.33	1.06	0.75, 1.49	1.28	0.82, 2.01
2000–3000	0.97	0.68, 1.39	1.24	0.92, 1.66	1.36	0.91, 2.03
3000–4000	0.96	0.65, 1.42	1.18	0.87, 1.61	1.10	0.70, 1.73
4000 and more	1.44	0.90, 2.29	1.55^*^	1.06, 2.26	1.53	0.84, 2.79
Prefer not to answer	0.61^*^	0.39, 0.95	0.84	0.60, 1.17	0.92	0.55, 1.54
Occupation						
Manual workers	Ref	Ref	Ref	Ref	Ref	Ref
Farmers	0.97	0.27, 3.50	1.43	0.51, 3.97	0.36	0.04, 3.26
Self‐employed and entrepreneurs	1.69	0.93, 3.09	1.04	0.60, 1.81	0.85	0.42, 1.74
Senior executive professionals	1.99^**^	1.20, 3.28	1.58^*^	1.04, 2.42	0.97	0.54, 1.75
Middle executive professionals	1.22	0.79, 1.89	1.31	0.92, 1.88	0.81	0.51, 1.28
Employees	1.29	0.85, 1.96	1.35	0.95, 1.92	1.05	0.69, 1.61
Retirees	2.24^**^	1.25, 4.01	1.36	0.84, 2.19	0.44^*^	0.20, 1.00
Never worked and others	1.20	0.74, 1.94	1.33	0.88, 2.02	0.54^*^	0.31, 0.95
Education						
High school	Ref	Ref	Ref	Ref	Ref	Ref
No diploma	2.62^***^	1.69, 4.07	0.80	0.52, 1.21	0.91	0.51, 1.62
Vocational secondary	1.39^*^	1.01, 1.90	0.96	0.74, 1.24	0.93	0.65, 1.33
High school+2	0.88	0.63, 1.24	1.03	0.80, 1.34	0.79	0.54, 1.18
High school+3	0.57^*^	0.35, 0.91	1.18	0.85, 1.64	0.97	0.58, 1.63
High school+4 or more years	0.84	0.53, 1.35	1.49^*^	1.04, 2.13	1.03	0.59, 1.82
COVID‐19 worry						
0 to 3	Ref	Ref	Ref	Ref	Ref	Ref
4–6	0.47^***^	0.32, 0.68	0.72^*^	0.55, 0.96	1.18	0.85, 1.65
7–8	1.57^**^	1.19, 2.06	1.86^***^	1.49, 2.32	0.97	0.67, 1.39
9–10	1.92^***^	1.33, 2.77	2.43^***^	1.81, 3.26	0.75	0.44, 1.29
Do not know	1.02	0.55, 1.87	0.77	0.45, 1.32	0.53	0.24, 1.17
COVID‐19 vaccine hesitancy						
Q1 (low)	Ref	Ref	Ref	Ref	Ref	Ref
Q2	1.22	0.79, 1.90	0.69^*^	0.51, 0.94	1.53	0.43, 5.42
Q3	1.17	0.77, 1.76	0.41^***^	0.30, 0.55	1.41	0.44, 4.54
Q4	0.96	0.64, 1.44	0.15^***^	0.11, 0.20	4.67^**^	1.63, 13.4
Q5 (high)	0.37^c^	0.23, 0.59	0.04^***^	0.03, 0.06	15.0^***^	5.27, 42.6
Party identification						
Centre	Ref	Ref	Ref	Ref	Ref	Ref
Far left	1.93^**^	1.26, 2.96	0.73	0.51, 1.04	0.79	0.42, 1.48
Left	1.03	0.58, 1.83	0.69	0.44, 1.06	0.42	0.15, 1.17
Green party	0.68	0.36, 1.26	0.76	0.50, 1.18	0.53	0.22, 1.26
Right	0.88	0.49, 1.59	0.71	0.46, 1.08	0.81	0.35, 1.88
Far right	0.99	0.65, 1.51	0.60^**^	0.43, 0.83	0.81	0.45, 1.48
No partisan proximity	0.54^**^	0.35, 0.84	0.45^***^	0.33, 0.62	0.49^*^	0.27, 0.92
Other	1.68	0.87, 3.25	0.62	0.34, 1.12	0.74	0.28, 1.94
Political trust						
Q1 (low)	Ref	Ref	Ref	Ref	Ref	Ref
Q2	1.14	0.79, 1.63	1.09	0.83, 1.44	0.82	0.59, 1.13
Q3	1.19	0.83, 1.71	1.16	0.88, 1.53	0.65^*^	0.44, 0.96
Q4	1.57^*^	1.06, 2.32	1.24	0.91, 1.68	0.49^**^	0.29, 0.84
Q5 (high)	2.75^***^	1.87, 4.06	1.24	0.90, 1.72	0.54	0.29, 1.03
Political sophistication						
Q1 (low)	Ref	Ref	Ref	Ref	Ref	Ref
Q2	1.13	0.80, 1.59	1.23	0.93, 1.61	1.12	0.78, 1.63
Q3	1.91^***^	1.34, 2.71	1.63^**^	1.22, 2.18	1.15	0.76, 1.75
Q4	2.14^***^	1.44, 3.19	2.74^***^	2.01, 3.74	1.11	0.70, 1.76
Q5 (high)	1.82^**^	1.21, 2.76	1.97^***^	1.42, 2.74	1.23	0.77, 1.97

Abbreviation: RRR = relative risk ratios.

^*^ *p* < 0.05.

^**^ *p* < 0.01.

^***^ *p* < 0.001.

## 4 Results

### 4.1 Overview of Ordinary Contributions to the COVID‐19 Vaccination Campaign

For each of our five social circles, respondents could either only contribute positively (i.e., trying to convince people to get the vaccine), only contribute negatively (i.e., trying to convince people of the opposite), do both or not contribute at all.

Looking at the patterns of contribution in all social circles taken together, we observe that 27.6% of the sample never tried to convince people either way in any social circle (see Table 1). These *noncontributors* represent the second largest group in our sample. The largest group is comprised of those who only contributed positively, which represents around half of the sample (*the allies*, 49.5% of the sample). Opposite them, those who only contributed negatively constituted the smallest group with only 9% of the sample (*the opponents*). Importantly, this group is smaller than that of the respondents who engaged in both types of contributions (*the versatile*, 13.9%).

### 4.2 Overall Association Between Independent Variables and Patterns of Contribution to COVID‐19 Vaccination

To start understanding what differentiates these different groups, we turn to our Type II analysis, which allows us to compare the explanatory power of our different variables across the board (Supporting Information Table S5). We observe that all variables have a significant effect on our outcome (*p* < 0.001) except occupation and income. The most important variables by far relate to dispositions towards health. They are COVID‐19 vaccine hesitancy (LR chi‐squared of 866***), COVID‐19 worry (130***) and age (95***). However, our political variables also appear to be important determinants (party identification: 92***, political trust: 59***, political sophistication: 58***).

To test our hypotheses, we must compare more precisely the factors that distinguish each of our four groups from the others.

### 4.3 Noncontributors to COVID‐19 Vaccination

We start with a focus on *noncontributors*. In our bivariate analyses (see Table 1), we observe strong and significant differences in the propensity to be a *noncontributor* across almost all our political variables (Hypotheses 2.1–2.3). The less politically sophisticated and the nonpartisan are much more likely to be *noncontributors*, whereas respondents who feel close to parties at the far right and respondents with a high level of political distrust are only slightly more likely to be among them. For instance, 43.7% of respondents in the least politically sophisticated quintile are *noncontributors*, whereas this is the case for only 19.4% of the most politically sophisticated. We observe slight differences according to age (21% of respondents aged 65 and over are among the *noncontributors* vs. 26%–33% in the other age categories), but no over‐representation of men in this group. We can also note that we observe an important social gradient in the propensity to contribute: The wealthiest and those with a higher level of educational attainment are rarely *noncontributors*. More importantly, differences according to the level of COVID‐19 vaccine hesitancy are the strongest, with the more hesitant being much more likely to avoid trying to influence others. For instance, 47.6% of respondents in the most hesitant quintile belong to this group, and so do 41.5% of respondents with the lowest COVID‐19 worry score (Table 1).

The multinomial regression shows that groups with positive contributions (*allies* and *versatiles*) differ the most from *noncontributors* (Table 2). By contrast, the profile of *opponents* appears to be quite similar to that of *noncontributors*, except for the decisive variable of vaccine hesitancy. This conclusion is based on the relative risk ratios (RRR, which are the exponentiated regression coefficients, as is standard practice for multinomial models; see Fitzhugh et al. 2023), which indicate the largest contrasts between these groups. This differentiation is mainly attributable to dispositions towards health. Individuals who are most worried about COVID‐19 (compared with those in the reference category reporting the lowest level of worry) are much more likely to be *allies* or *versatiles* rather than *noncontributors* (RRR = 2.43*** and 1.92***, respectively). Conversely, those exhibiting the highest level of COVID‐19 vaccine hesitancy (compared with the reference category reflecting the lowest hesitancy) are much less likely to be *allies* or *versatiles* than *noncontributors* (RRR = 0.04*** and 0.37***). In particular, individuals with a high level of political sophistication (compared with those in the reference category reporting the lowest level of sophistication) are more likely to be *allies* or *versatiles* than *noncontributors* (for Q4, RRR = 2.74*** and 2.14***). Conversely, those who do not feel close to any party (compared with centrists, the reference category) are much less likely to fall into the *allies* or *versatiles* groups rather than the *noncontributors* group (RRR = 0.45*** and 0.54**). Furthermore, individuals with a high level of political trust (compared with those reporting the lowest trust) are much more likely to be *versatiles* rather than *noncontributors* (RRR = 2.75***), whereas respondents who feel closest to the far right are significantly less likely to be *allies* than *noncontributors* (RRR = 0.60**). It should also be noted that women (compared with men) are more likely to be *allies* than *noncontributors* (RRR = 1.36**). Older respondents (compared with the youngest respondents) were much more likely to be *versatiles* than *noncontributors*. The differences between *opponents* and *noncontributors* mainly concern vaccine hesitancy: Individuals with higher vaccine hesitancy (compared to those with lower hesitancy) are significantly more likely to be *opponents* rather than *noncontributors* (RRR = 15***). Conversely, those with higher levels of political trust (compared to the lowest level) are substantially less likely to be *opponents* than *noncontributors* (for Q4, RRR = 0.49**). Finally, individuals aged 35 to 64 (compared to those aged 18–24) are less likely to be *opponents* than *noncontributors* ([35–49] RRR = 0.48** and [50–64] RRR = 0.41***).

This focus on *noncontributors* mainly speaks to hypotheses H1.3, which expected women to be less present in this group than in any other group, and H2.3, which expected this to be the case for the least politically sophisticated. H1.3 was not confirmed—gender differentiating only *noncontributors* from *allies*—whereas the support for H2.3 was greater but not complete—political sophistication differentiating this group only from the groups with positive contributions but not from *opponents*. The results concerning the role of vaccine hesitancy also point towards a nuanced approach to our hypotheses. With H1.1, we expected positive contributions to be associated with those more vulnerable and concerned with COVID‐19, whereas H1.2 expected the more hesitant to engage more in negative contributions. An important result is, therefore, that a sizeable segment of vaccine‐hesitant respondents and of respondents who were not worried about COVID‐19 did not voice their judgement around them. These results suggest a more complex interplay between political and health dispositions, especially the need to investigate the conditions under which vaccine hesitancy translates into negative contributions rather than noncontribution (see the last segment of the Results section).

This is also what we can draw from the comparisons between the groups who contributed to the COVID‐19 vaccination, to which we will now turn.

### 4.4 What Distinguishes *Allies*, *Opponents* and *Versatiles*?

In the multivariate analyses, those who only contributed negatively (*opponents*) display very different characteristics from the two groups who contributed positively (Supporting Information Table S2). As expected, individuals who are most vaccine‐hesitant (compared with those with the lowest level of hesitancy) are less likely to be *allies* or *versatiles* than *opponents* (RRR = 0.00*** and 0.03***, respectively). Similarly, those who are most worried about COVID‐19 (compared with those who report being the least worried) are more likely to be *allies* or *versatiles* than *opponents* (RRR = 3.22*** and 2.55**). This pattern also holds for individuals with a high level of political trust (RRR = 2.28* and 5.05***) and, less markedly, for those with a high level of political sophistication (for Q4, RRR = 2.46*** and 1.93*) (compared with respondents with the lowest levels). This is reflected in the bivariate analyses. For instance, 74.7% of *opponents* belong to the two quintiles with the lowest political trust, 93.5% to the two most hesitant quintiles and 70% display low or average levels of COVID‐19 worry (vs. 42.3% of the sample). However, we did not find the expected differences according to party identification in the multivariate analyses, even though respondents close to the far right are heavily over‐represented in this group (43.5% of *opponents* vs. 23.4% of the sample). On the contrary, we even found that respondents closer to parties at the far left were more likely to be in the less negative group of *versatiles* than among *opponents*. The multinomial regression also suggests that individuals aged 65 or more (compared with those in the reference category aged 18–24) are much less likely to be *versatiles* rather than *opponents* ([65–74] RRR = 0.15*** and [75 and over] RRR = 0.10***). However, the bivariate analyses show minute differences on this point, and older respondents are under‐represented among *opponents* compared to the composition of the overall sample.

This can partly be explained by the specificities of the *versatiles*. This group is the youngest of all (38.3% aged 34 or less vs. 23.6% of the sample; see also the multivariate regression in Supporting Information Table S3). It is also the one with the highest proportion of respondents from the far left (31.9% of this group), and members of this group are much more likely to have a very high degree of political trust. They are much less vaccine‐hesitant than *opponents* but much more than *allies*. They are also less likely to have a low level of COVID‐19 worry than the former.

It surfaces from these comparisons that *allies* are the group that fits the most with expectations, especially when we look at the bivariate analyses. We find an over‐representation in this group of the least vaccine‐hesitant (63.2% of *allies* in the two lowest quintiles), of respondents with a high level of COVID‐19 worry (61.9% of *allies* vs. 54.7% of the sample), of the politically sophisticated (51.2% in the two highest quintiles), of respondents close to parties at the centre (26.9% vs. 17.9% of the sample) and of respondents aged 65 or more (37.9% vs. 28.3% of the sample). The multinomial regression largely confirms this description while underlining the fact that the main specificity of this group rests in its vaccine confidence. We can also note that the bivariate analyses underline a strong social gradient in the composition of this group: The poorest and those with the lowest levels of educational attainment are very under‐represented among *allies*.

This comparative description of the three groups who engaged in ordinary contributions to COVID‐19 vaccination will probably not have appeared as clear as was hoped. This is a result in and of itself. As for noncontribution, what determines the type of contribution does not seem to neatly follow expectations regarding the role of dispositions towards health and politics. H1.2, which expected negative contribution to be more prevalent among the more vaccine‐hesitant, is the most substantiated. Both in the bivariate and multivariate analyses, *opponents* are the most vaccine‐hesitant group, *allies* are the least and *versatiles* are in between the two. We can also evoke the fact that 81.8% of respondents in the least hesitant quintile are *allies*, whereas less than 8% of them contributed negatively. H1.1, which expected elderly respondents and those most worried about COVID‐19 to engage more in positive contributions, is also largely corroborated. Around 60% of respondents with a level of COVID‐19 worry of 7 or more were *allies*, whereas respondents with a low level of COVID‐19 worry were much more likely to be *opponents*. However, it must be noted that *allies* and *versatiles* did not differ significantly on this front. A significant proportion of those worried about COVID‐19 also dissuaded some people around them from getting vaccinated. The effect of age seems even more subtle. Although we found the expected over‐representation of the young among *opponents* and of the older among *allies*, we also found that a significant proportion of the young also contributed positively (*versatiles*). These results underline even more strongly what was already striking in the first results we presented: The factors favouring engagement in negative contributions do not preclude also engaging in positive ones.

A similar conclusion applies to our hypotheses focused on political dispositions. H2.1 expected a clear separation between respondents at the far end of the political spectrum and those closer to the centre. The bivariate analyses tend to support this across the board: Respondents close to parties at the centre and to their partners at the left and right are very likely to be among *allies* (> 66%) and very unlikely to be among *opponents* or *versatiles* (< 15%), whereas respondents close to parties at the far right and far left are unlikely to be among *allies* (< 37%). However, we found that these differences often become insignificant when controlling for other variables such as vaccine hesitancy. We also found a clear demarcation between identifying with the far left and the far right, the former being much more associated with being *versatile*, whereas the latter are more likely to only contribute negatively. The findings relative to the role of political trust (H2.2) lead to a similar conclusion. We do find a simple opposition between *allies*, who display a high political trust, and *opponents*. However, we also found a high likelihood of being *versatile* among respondents with a high level of political trust when controlling for other factors. We also found that political sophistication does not only play a role in whether people engage in any kind of contribution (H2.3, discussed in the previous section). *Opponents* distinguished themselves from both *allies* and *versatiles* by their tendency to be less politically sophisticated.

## 5 Discussion

### 5.1 Main Results

Drawing on a large survey, we explored the patterns of ordinary contributions to COVID‐19 vaccination in France. We found that very few respondents only tried to dissuade people from getting vaccinated (9%). Around half only contributed positively, with an important minority who either engaged in both types of contributions or abstained from trying to influence others altogether. Using hypotheses grounded in the sociology of health and in political sociology, we showed that respondents’ propensity to try to influence others and the directions in which they did so was influenced primarily by their attitudes towards the vaccine and the disease but also, to a significant extent, by their political dispositions. We found, as expected, that (a) *opponents* were very vaccine‐hesitant, did not worry about COVID‐19 very much, were younger, felt closest to radical political parties and had little political trust, whereas (b) *allies* were the opposite. But we also found that a significant proportion of the vaccine‐hesitant did not try to influence others or contributed both negatively and positively. We also found that gender did not have a strong effect on these dispositions after controlling for other factors. We expected political sophistication to facilitate engagement in any kind of contribution but found that it only seemed to have this effect among the vaccine confident. Our results confirm that ordinary contributions to vaccination are to a large extent a form of political action. However, our guiding hypotheses were only partly corroborated, which calls for a more in‐depth exploration of the interplay between these dispositions towards health and politics and a discussion of the implications of our findings.

### 5.2 On the Influence of the Vaccine‐Hesitant

A first input from our study is that we show that people who at one time or another advised someone against COVID‐19 vaccination also did the opposite in almost 50% of cases. This result probably reflects the conditional, subtle and evolving nature of negative attitudes towards vaccines, well documented in the literature on vaccine hesitancy (Dubé et al. 2021; Enkel et al. 2018; Larson et al. 2022). In the present case, this “both ways” form of contribution may reflect differences in appreciation of the benefit–risk balance of COVID‐19 vaccination (“the vaccine is useful for the vulnerable, but not for healthy people like me”) and an improvement of attitudes towards COVID‐19 vaccines over time (Ward, Privault, et al. 2024).

This versatile contribution to COVID‐19 vaccination among many of the hesitant is likely to limit the potential knock‐on effects of their negative personal attitudes towards vaccines. It should also be noted that the people who tried to convince others not to get vaccinated tended to be on the lower end of the social hierarchy. This reflects the well‐documented social inequalities in attitudes to vaccines in France and many other countries (Dubé et al. 2021; Larson et al. 2022) (Ward, Privault, et al. 2024), but it may also help to explain them. People in these social groups are more likely to be confronted with critical discourse on vaccines emanating from their family, friends and colleagues. This first set of results underlines the importance of placing relationships to vaccines in their context of formation and of thinking of them in relational terms (Are et al. 2024; Brunson 2013; Facciani et al. 2023).

### 5.3 Political Empowerment, the “Antivaccine” Label and Conflict Avoidance

Our main result is that there are significant discrepancies in respondents’ propensity to translate their personal beliefs into attempts to influence others. The hesitant are much more likely to be *noncontributors* than the nonhesitant. An explanation for this discrepancy lies in the symbolic status or norm associated with vaccination in contemporary societies. Vaccine critics and the nonvaccinated are constantly denounced as antiscience, conspiracy theorists and irresponsible (Court et al. 2021; Hausman 2019; Vanderslott 2019), especially in France (Ward et al. 2019). This situation may dissuade many hesitant people from speaking their minds for fear of creating tension or of social sanctions (Bor et al. 2023; Heyerdahl et al. 2022; Wagner and Eberl 2024). Our study also shows that this lack of engagement in ordinary contributions to vaccination is not restricted to the hesitant. It is possible, then, that this conflictual nature of vaccination dissuaded a wider audience from engaging with this theme.

This phenomenon can also have been exacerbated by the fact that the topic of vaccination has become political. The political nature of debates over vaccination does not only have implications regarding how people form their opinion on the subject. It also affects whether they wish to talk about it with the people around them. Indeed, many people try to avoid talking about political issues in their everyday life because of their conflictual nature but also because of a sense of lack of competence and interest in the subject (Eliasoph 1998; Gamson 1992). This interpretation is buttressed by our finding that political dispositions traditionally associated with political conflict avoidance are significant determinants of respondents’ propensity to engage in ordinary contributions to COVID‐19 vaccination. The effect of political distrust, nonpartisanship and low political sophistication on dispositions to voice one’s beliefs about vaccines can be understood in the following way. During the COVID‐19 epidemic, vaccination became one of the main topics in the news. It was, therefore, caught up in the kind of interactions that take place around political news, and proficiency in talking about politics could have become perceived as a relevant skill to talk on this subject. One consequence of this could be that, for example, the most politically sophisticated feel legitimate talking about health issues.

By showing that political sophistication plays a role in the propensity to try to influence the vaccination behaviours of others even after controlling for attitudes to vaccines and perception of the disease, our work extends the rare studies that have examined the effects of this dimension on health behaviours in general (Mattila et al. 2017) and vaccination in particular (Blank and Shaw 2015; Enders and Uscinski 2021; Joslyn and Sylvester 2019) (Ward, Cortaredona, et al. 2024). These studies have tended to focus on the fact that political sophistication is generally associated with other forms of social capital, such as education, which allow people to identify health‐related resources and better assess the quality of information on this topic. Our study highlights another potential form of transfer of political dispositions to other sectors through the question of self‐empowerment—the feeling that one is legitimate to form and express an opinion on any issue.

### 5.4 Politicisation of Vaccines and the Legitimisation of Vaccine Criticism

We also found that, even after controlling for vaccine hesitancy and concern regarding COVID‐19, the factors largely associated with attitudes to vaccines in France also independently bear on the propensity to influence others (age, gender, income, partisanship, political trust, political sophistication; Ward, Cortaredona, et al. 2024; Ward, Privault, et al. 2024). Given the fact that people tend to socialise more with people with similar social and political backgrounds, this could reflect variation in the strength of the norm to vaccinate in their social circles. This is another implication of the politicisation of debates over vaccines. By endorsing vaccine‐critical arguments, mainstream political parties are not only likely to elicit doubt among their followers, but also to shift the symbolic status of vaccine criticism. This shift was well theorised by Daniel Hallin, who speaks of the circulation of topics of public discussion from “the sphere of deviance” to the sphere of “legitimate controversy” (Hallin 1986), and by Uscinski et al., who speak of a “mainstreaming of conspiracy theories” (Uscinski et al. 2021). Starting at the political extremes, vaccine criticism could therefore be progressively becoming an opinion just like another: one that people can disagree with but which can be voiced publicly because it does not expose the person to shaming or ridicule.

### 5.5 Limitations

Our study has several limitations. Firstly, it has the usual limits of cross‐sectional studies, well exposed by Wang and Cheng (2020). In particular, we cannot establish a temporal sequence between exposure and outcome, opening the possibility of reverse causality. Although our results reveal important and significant patterns, prospective cohort studies are necessary to validate these associations and infer causality. Secondly, we asked respondents to reflect on their behaviour during the year and a half prior to the survey, creating the possibility of some recall bias. Thirdly, the questionnaire was self‐administered online using quota sampling rather than via face‐to‐face interviews among a random sample. Online studies can lead to under‐representation of social groups with limited internet access and the weakly digitally literate. However, they have the advantage of limiting desirability bias in responses (Kreuter et al. 2008), which is a crucial issue on a topic heavily imbued with norms such as vaccination. It remains that, in addition to biases in who is contacted to participate in the study, quota sampling among a panel makes it difficult to clearly identify selection biases, including self‐selection by respondents. Finally, our study is declarative, and we could not observe actual behaviours. Our findings should therefore be compared to those of studies drawing on ethnography and social media analysis.

### 5.6 Conclusion

In this paper, we wished to draw attention to *ordinary contributions to public policies* in the domain of health by investigating the differences between those who kept their opinions on COVID‐19 vaccination to themselves and those who actively tried to influence others, as well as between public supporters and opponents of this policy. Beyond vaccination, we believe that the study of *ordinary contributions* can advance our understanding of the way contemporary health experiences are imbued with collective norms, embedded in social interactions and group sociabilities and shaped by the emergence of politicised public debates.

## Author Contributions

**Hugo Touzet:** conceptualization, investigation, writing – original draft, methodology, validation, visualization, writing – review and editing, formal analysis, project administration, data curation. **Benoit Giry:** conceptualization, writing – review and editing, methodology, validation, visualization, formal analysis, supervision. **Jeremy K. Ward:** conceptualization, investigation, funding acquisition, writing – original draft, validation, writing – review and editing, project administration, supervision, resources.

## Funding

This study has been labelled as a National Research Priority by the National Orientation Committee for Therapeutic Trials and other studies on COVID‐19 (CAPNET, project ICOVAC‐France: 0344). The investigators would like to acknowledge ANRS | Emerging Infectious Diseases for their scientific support and the French Ministry of Health and Prevention and the French Ministry of Higher Education, Research and Innovation for their funding and support. It has also benefited from funding from the Agence nationale de la recherche (ANR‐20‐COVI‐0035‐01, ANR‐22‐CE36‐0015‐01). The funding sources had no role in the design of the study, the analysis of the data or the writing of the paper.

## Ethics Statement

The methodology of the study was reviewed and approved by the Institutional Review Board of the Inserm (#21–770).

## Conflicts of Interest

The authors declare no conflicts of interest.

## Supporting information


Supporting Information S1

## Data Availability

The data that support the findings of this study are available from the corresponding author upon reasonable request.
